# Acute kidney injury in a patient with nontuberculous mycobacterial infections: a case report

**DOI:** 10.1186/1757-1626-2-83

**Published:** 2009-01-23

**Authors:** Zachary Z Brener, Igor Zhuravenko, Michael Bergman

**Affiliations:** 1 Department of Medicine, Nephrology Division, Beth Israel Medical Center, University Hospital of Albert Einstein Medical Center, 350 East 17th Street, 18th Floor, New York, NY, 10003, USA; 2 Department of Medicine, Ocean Medical Plaza, Brooklyn, NY, 11235, USA; 3 Department of Medicine, Campus Golda-Hasharon, Rabin Medical Center, Affiliated with Tel-Aviv University, Petah-Tikva, Israel

## Abstract

Nontuberculous mycobacterial infections are an increasingly recognized cause of chronic lung disease in both immunocompromised and immunocompetent patients. Pre-existing lung disease, alcohol abuse, diabetes mellitus, malignancy, and smoking have been identified as important risk factors in nontuberculous mycobacterial infections, with only few cases of Nontuberculous mycobacterial infection in renal failure patients, mostly on peritoneal dialysis. However, acute kidney injury associated with atypical mycobacterial infection is a very rare clinical event. To our knowledge, the present patient is the first case of acute kidney injury in a patient with documented nontuberculous mycobacterial infection. Our case is also a first report of *Mycobacteria avium *complex and *Mycobacteria gordonae *isolated simultaneously from individual patient with nontuberculous mycobacterial disease.

## Introduction

Nontuberculous mycobacterial (NTM) infections are an increasingly recognized cause of chronic lung disease in both immunocompromised and immunocompetent patients [1,2]. Pre-existing lung disease, alcohol abuse, diabetes mellitus, malignancy, and smoking have been identified as important risk factors in NTM [3], with only few cases of NTM infection in renal failure patients, mostly on peritoneal dialysis [4]. However, acute kidney injury (AKI) associated with NTM infection is a very rare clinical event. To our knowledge, the present patient is the first case of AKI in a patient with documented NTM infection. This is also the first reported case of *M. avium *complex and *M. gordonae *isolated simultaneously from individual patient with NTM disease.

## Case presentation

**Figure 1:**
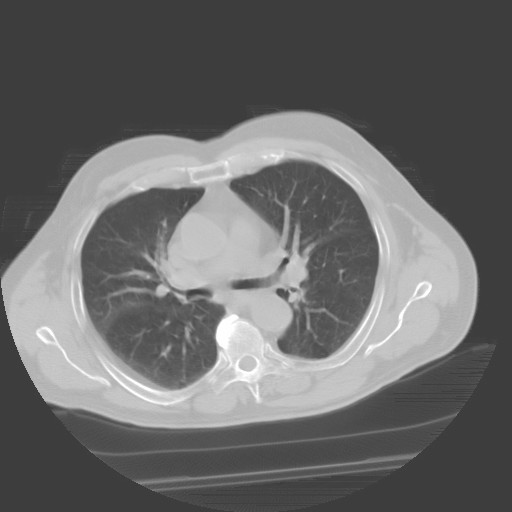
**CT scan of the chest showing right lower lobe scarring and left upper lobe small nodules with focal pleural thickening**.

A 72-year-old man with known history of stage 3 chronic kidney disease, hypertension, coronary artery disease, hyperlipidemia presented with a 3-days history of low-grade fever, productive cough, dyspnea, night sweats and fatigue. Patient was a non-smoker and did not consume alcohol. His kidney disease was likely due to renovascular or/and chronic atheroembolic disease with baseline serum creatinine level of 1.7 mg/dl, giving him an estimated glomerular filtration rate of 50 mL/min/1.73 m^2^. His medications included metoprolol, isosorbide mononitrate, gabapentin, aspirin, candesartan, tamsulosin, atorvastatin. On examination, he was alert, and not in distress. His blood pressure was 140/80 mm Hg without orthostatic changes and temperature 100.3°F. The remainder of the examination was unremarkable except of few wheezes over both lung fields. He did not have pedal edema. Serum sodium was 145 mEq/L, potassium 4.8 mEq/L, urea nitrogen 34 mg/dl, creatinine 1.7 mg/dl and glucose 130 mg/dl. Hemoglobin was 12.1 g/dL, white blood cells 5.2 × 10^9^/L and platelets 208 × 10^9^/L. Treatment with 250 mg of azithromycin daily was given for six days without clinical improvement. A chest radiograph showed small left apical nodules and computed tomography (CT) scan of the chest demonstrated right lower lobe scarring and left upper lobe small nodules with focal pleural thickening (Figure 1). Three weeks later, serum creatinine rose to 2.6 mg/dl and urea to 48 mg/dl. Urine volume never decreased; urinalysis showed trace proteinuria, without glucose or blood. Urine sediment did not show red blood cells, white blood cells or casts. Urine sodium was 35 mmol/L. Patient completed 2 weeks treatment with 500 mg of levofloxacin, with some clinical improvement. Six weeks after initial presentation, cultures of two sputum samples were reported to be positive for *Mycobacterium gordonae *and *Mycobacterium avium *complex (MAC) by DNA probe and subsequently the positive diagnosis of pulmonary nontuberculous mycobacterial infection was made. The patient rejected long-term triple therapy, given his mild symptoms and a concern about treatment adverse effects. The patient has remained well throughout a 2 months period of close clinical observation; he has been afebrile and his serum creatinine level returned to baseline of 1.7 mg/dl.

## Discussion

Pulmonary disease due to NTM typically occurs in patients with impaired cellular immunity or chronic lung disease [3]. Recently, there has been an increase in the number of reports of pulmonary disease caused by NTM occurring in otherwise healthy individuals, and the *M. avium *complex (MAC), and *M. kansasii *account for most of the pathogens involved [1]. Signs and symptoms of NTM lung disease are often variable and nonspecific. Patients frequently present with chronic cough, productive sputum, and fatigue [2]. The radiographic criteria required are the presence of infiltrates, cavitations, or nodules at chest radiography, and/or multiple small nodules or multifocal bronchiectasis at CT of the chest [2].

MAC is the most commonly isolated and the most clinically important pulmonary NTM pathogen, and includes the two species *M. avium *and *M. intracellulare *[5]. The fact that they are distinct has no clinical value for individual patients, however, and they are generally not differentiated. To our best knowledge, simultaneous isolation of both MAC and *M. gordonae *from respiratory secretion of the same patient has never been described in a literature.

Coexisting medical conditions have been identified by studies throughout the world as important risk factors, with only few cases of NTM infection in renal failure patients, mostly on peritoneal dialysis [4]. An important role of infection-related mediator mechanisms in the genesis of AKI has been well recognized. However, AKI associated with NTM infection is a very rare clinical event. To our knowledge, the present patient is the first case of AKI in a patient with documented NTM infection. The chronological sequence of renal failure and recovery in the context of NTM infection raises a possibility that the renal injury might be at least partly due to direct infection and/or immune-mediated damage.

## Conclusion

Because of the increasing number of immunocompromised patients, immigrants, refugees, patients in congregate setting, and patients with drug-resistant disease, it is possible that renal involvement will be recognized among the presentations of NTM infection. Physicians should be aware of the possibility of NTM infection presenting with AKI, and our case report may help to elucidate the mechanisms of renal impairment in NTM infection. Increased number of cases of NTM disease caused by more than one pathogen can be also expected.

## Consent

Written informed consent was obtained from the patient for publication of this case report and accompanying images. A copy of the written consent is available for review by the Editor-in-Chief of this journal.

## Competing interests

The authors declare that they have no competing interests.

## Authors' contributions

ZZB participated in the sequence alignment, explored all possible sources for the references and drafted the definite version of this manuscript. IZ participated in the design of the study. MB conceived of the study, and participated in its design and coordination and helped to draft the manuscript. All authors read and approved the final manuscript.
